# Automated and Online Characterization of Adherent Cell Culture Growth in a Microfabricated Bioreactor

**DOI:** 10.1177/2211068214529288

**Published:** 2014-10

**Authors:** Nicolas Jaccard, Rhys J. Macown, Alexandre Super, Lewis D. Griffin, Farlan S. Veraitch, Nicolas Szita

**Affiliations:** 1 Department of Biochemical Engineering, University College London, London, UK; 2 Centre for Mathematics and Physics in the Life Sciences and Experimental Biology, University College London, London, UK; 3 Department of Computer Science, University College London, London, UK

**Keywords:** image processing, adherent cell culture, microreactors, stem cell growth kinetics, microfluidics

## Abstract

Adherent cell lines are widely used across all fields of biology, including drug discovery, toxicity studies, and regenerative medicine. However, adherent cell processes are often limited by a lack of advances in cell culture systems. While suspension culture processes benefit from decades of development of instrumented bioreactors, adherent cultures are typically performed in static, noninstrumented flasks and well-plates. We previously described a microfabricated bioreactor that enables a high degree of control on the microenvironment of the cells while remaining compatible with standard cell culture protocols. In this report, we describe its integration with automated image-processing capabilities, allowing the continuous monitoring of key cell culture characteristics. A machine learning–based algorithm enabled the specific detection of one cell type within a co-culture setting, such as human embryonic stem cells against the background of fibroblast cells. In addition, the algorithm did not confuse image artifacts resulting from microfabrication, such as scratches on surfaces, or dust particles, with cellular features. We demonstrate how the automation of flow control, environmental control, and image acquisition can be employed to image the whole culture area and obtain time-course data of mouse embryonic stem cell cultures, for example, for confluency.

## Introduction

Many primary cell lines derived from animals must attach to a substrate to maintain viability and phenotype.^1,2^ Consequently, drug toxicity tests, vaccine development, and cell therapies rely significantly on well-developed and robust adherent cell culture processes. However, the significant advances in process control of the past two decades in biotechnology mostly occurred for suspension culture systems, for example, for the production of recombinant therapeutics.^3^ The ability to continuously monitor key process variables was paramount in the development of these robust processes. Thus, the development of novel approaches enabling monitoring capabilities comparable to that of suspension culture systems may facilitate further growth in the applications relying on adherent cell culture.

Monitoring adherent cell cultures poses numerous challenges. Unlike with suspension cultures, the notion of a representative sample is rarely applicable to adherent cultures due to their inherent inhomogeneity. There is also a general lack of instrumentation as adherent cultures are usually carried out in disposable plastic vessels. As a consequence, most assays are based on techniques developed for suspension cultures that require the detachment of the cells prior to analysis and are thus limited to end-point analysis. This limitation not only prevents the determination of key characteristics that are only observable while cells are attached (e.g., confluency, morphology, distribution, expression patterns) but also precludes the generation of time-course data needed to quantify growth kinetics.

In contrast, light microscopy methods require neither cell detachment nor sampling. Indeed, visual inspection of culture vessels using phase contrast microscopy (PCM) enables the qualitative assessment of both growth and cell phenotype. When combined with automated image-processing methods, PCM was shown to enable the quantification of adherent cell culture characteristics such as confluency and morphology.^4–7^ Due to the nature of the image-processing algorithms employed, these methods are best suited to simple experimental setups where the phenotype and visual features of the studied cells remain relatively unchanged during the course of an experiment. Trainable segmentation methods, based on machine learning classifiers, are often better suited for complex experimental scenarios such as co-cultures.^8^ In addition, such methods were previously shown to offer a high degree of flexibility and are expected to enable the monitoring of cell phenotypes that undergo significant variations during culture.^9^

For any application, the accuracy and precision of the image-processing data will be influenced by the fraction of the culture area that can be imaged with reasonable effort (i.e., how much of the culture can essentially be sampled). The higher this fraction is, the less error will be inherent in the resulting measurements.^4^ For this reason, performing adherent cell culture at the microscale offers a significant advantage. The small size of culture chambers in microculture systems enables the imaging of the whole culture area in a minimum period of time. Moreover, the combination of microfluidic concepts with adherent cell culture enables the automation of essential culturing steps such as medium exchange and fine control over the microenvironment of the cells.^8,10–16^ Finally, microculture systems generally require fewer resources for a given process, allowing experimentation at a reduced cost.

In this contribution, we present the integration of a previously described microfabricated device^8^ with automated image acquisition and processing routines. Requirements and strategies for intermittent and continuous imaging are described. In addition to previously reported human embryonic stem cell colony monitoring capabilities, the image-processing algorithm was further improved to enable the monitoring of long-term on-chip mouse embryonic stem cell cultures. In both cases, cell proliferation was characterized at a population level using confluency while cellular object tracking helped gain an insight into the response of colonies to continuous perfusion.

## Material and Methods

### Microfabricated Culture Device

The previously described microfabricated culture device^8^ is a modular design assembled from a combination of disposable and reusable components. Rigid polycarbonate frames compress a poly(dimethylsiloxane) (PDMS) chip against a microscope slide, forming a reversible seal. The PDMS chip contains microfluidic channels to perfuse media uniformly through a culture chamber. Cells are grown directly on the microscope slide. The growth area of the culture chamber was 0.52 cm^2^. The culture chamber is directly accessible by removal of a polycarbonate lid. The polycarbonate lid defines the height of the culture chamber as 450 µm and seals the chamber by compression of a PDMS gasket. For the human embryonic stem cell (hESC) culture experiments, the microscope slide used was made of Tissue Culture Polystyrene (16004; Nunc, Roskilde, Denmark). Perfusion of media was achieved using a syringe pump as previously described.^8^ For the mouse embryonic stem cell (mESC) cultures, the microscope slide used was made of glass. Perfusion was achieved using the pressure driven system previously described.^13^

### Human Embryonic Stem Cell Cultures

Human embryonic stem cells Shef-3 (< passage 70) were grown on a layer of mouse embryonic fibroblast (MEFs) feeder cells (< passage 5) following a protocol previously described.^8^ The sterile microfabricated bioreactor was assembled in a biosafety cabinet and seeded with MEFs (~15,000) before being transferred in a humidified CO_2_ (5%) incubator at 37 °C and incubated for 24 h to let the feeder cells settle down and attach. Then, medium was changed, and dissected hESCs colonies were seeded on top of the feeder layer and let to adhere for an additional 24 h in the incubator. The following day, a syringe pump was connected to the device and continuous perfusion at 300 µL/h was started for 2 days. Images of the culture chamber were manually taken each day by removing the device from the incubator and placing it on an inverted microscope (Inverted Microscope System TE2000; Nikon Ltd, Kingston upon Thames, UK). Images were acquired at a 10× magnification using a Fi-1 color camera (Nikon Ltd) and had a resolution of 1280 × 960 pixels (equivalent to a field of view ~1.2 mm^2^).

### Mouse Embryonic Stem Cell Cultures

E14tg2A mouse embryonic stem cells (< passage 50) were maintained in knock-out Dulbecco’s modified Eagle’s medium (DMEM) (10829; Gibco, Carlsbad, CA) supplemented with 15% (v/v) fetal bovine serum (FBS) (26140; Gibco), 1% (v/v) modified Eagle’s medium nonessential amino acids (11140; Gibco), 10% (v/v) Glutamax (35050; Gibco), 0.1 mM β-mercaptoethanol (31350; Gibco), and 10-6 U.L-1 Leukemia Inhibition Factor (LIF) (ESG1106; Millipore, Watford, UK).

The microfabricated bioreactor parts, perfusion reservoir, and tubing were autoclaved before being assembled in a biosafety cabinet. The chamber bottom was coated with gelatin (G1890; Sigma-Aldrich, Gillingham, UK), and the system was primed with growth medium. The device was then placed on the stage of an automated inverted microscope (Inverted Microscope System Ti-E; Nikon Ltd) inside a temperature-controlled cage incubator (H201; Okolab, Pozzuoli, Italy).

Cells were enzymatically harvested (Trypsin-EDTA T4049; Sigma-Aldrich) from T-25 flasks (Nunc EasyFlask 156367; ThermoScientific, Waltham, MA), spun down at 300 *g* for 3 min (Heraeus MultiFuge X3R; ThermoScientific), and resuspended in fresh growth medium. They were then manually counted using a hemocytometer and diluted in medium to reach the desired seeding density of 5 × 10^5^ cells/cm^–2^. The inoculum was seeded directly in the device chamber through the open lid and let in static culture for 3 h, to ensure that the cells properly adhered to the substrate. During that time, stage positions for phase contrast imaging of the whole culture area were manually recorded with a custom LabVIEW (National Instruments, Austin, TX) routine. Perfusion at 300 µL/h controlled by a pressure regulator (ITV0011-2BL-Q; SMC, Milton Keynes, UK) and automated imaging were then started. The course of the culture was monitored for 6 days and the system manually checked daily to detect any faults. Images were acquired at a 10× magnification using a Fi-1 color camera (Nikon Ltd) and had a resolution of 1280 × 960 pixels (equivalent to a field of view ~1.2 mm^2^).

### Automated Detection of Cellular Objects on PCM Images

Image processing was carried out using MATLAB (MathWorks, Natick, MA) to automate the detection and subsequent characterization of cellular objects on PCM images. A previously described machine learning–based approach was employed.^8^ In short, images were first converted to a gray-scale representation using the built-in *rgb2gray* function. Each pixel image was then classified as one of seven basic image features (BIFs) based on local symmetries.^17^ BIFs were computed at four scales (0.7, 1.4, 2.8, and 5.6). For each scale, local histograms of BIFs were computed in overlapping 25 × 25 windows so that each pixel was associated with a histogram. Histograms across the four scales for a given pixel were then concatenated. Each pixel of the original image was thus encoded as a feature vector containing 28 elements (seven-bin histograms at four scales). A random forest classifier^18^ was used to classify pixels as either background or cell based on their feature vectors. The classifier was both trained and validated using manually processed PCM images.

## Results and Discussion

### Integration of Image Acquisition and Processing with a Microfabricated Platform

**Figure 1.**
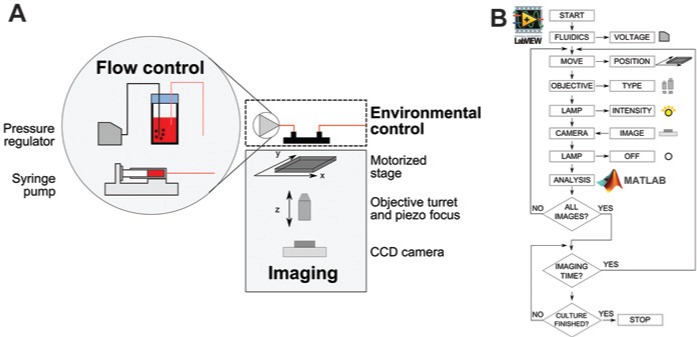
Schematic of the key components required for automated culture and monitoring of adherent cells in a microfabricated bioreactor. (**A**) Flow is controlled either by modulating the head pressure in a bottle containing the culture media or via a syringe drive. Temperature control is achieved using an on-stage incubator that houses the microfabricated bioreactor as well as the fluidics. A motorized stage is used together with a piezo focus system for imaging. (**B**) Schematic of a typical monitoring loop for the system. Automation of the fluidics and the imaging system is achieved using a LabVIEW routine while automated image processing was done using MATLAB.

The use of a microfabricated device as both a culture and imaging chamber requires the following key components (**Fig. 1A**): flow control for culture medium exchange, environmental control to maintain optimal temperature for growth, and an imaging system for monitoring. The implementation of these three components depends on the monitoring strategy. Intermittent monitoring allows the transport of the microfabricated device from its controlled environment (e.g., incubator) to the microscope for image acquisition. In contrast, online monitoring is achieved by having the device permanently positioned on top of a microscope stage and thus requires a suitable setup to maintain optimal growth conditions. Online monitoring offers obvious advantages, such as high image sampling rates, flexible monitoring schedules, reduced contamination risks (no need to transfer to and from an incubator), and a higher degree of automation (and thus reduced user interaction). Its main limitation lies in the number of devices that can be monitored at once with one microscope. To demonstrate the applicability of our imaging and automation routines, we tested both monitoring concepts.

For intermittent monitoring, the microfabricated device was kept in a standard cell culture incubator where both the temperature and gas atmosphere were controlled. A syringe pump was used to drive culture medium through the device. In contrast, for online monitoring experiments, the device was placed in an on-stage incubator for temperature control and the flow rate was controlled by varying the head pressure of a glass bottle containing the culture medium. This enabled the saturation of the liquid phase with oxygen and carbon dioxide, which were supplied using an external gas bottle. In addition, pressure-driven flow had a lower pulsatility compared with the flow generated using a syringe pump.^13^

Imaging was carried out using an automated microscope. As high-magnification objectives have a relatively small field of view compared with the area of the culture chamber (~1.20 mm^2^ for the 10× objective used in this study and 50 mm^2^, respectively), it is necessary to scan the culture chamber and acquire multiple images. This was accomplished using an encoded motorized *x*-*y* stage. If necessary, adjustment of a piezo *z* stage could be used to maintain accurate focus across multiple fields of view and over time.

Automation of the monitoring loop was achieved using LabVIEW (**Fig. 1B**). Virtual instruments controlling the individual functions of the microscope (e.g., motorized stage, objective turret) as well as the digital camera were interfaced with dynamic linked libraries made available by the manufacturer. A graphical user interface (GUI) allowed full manual control of the microscope and displayed a continuous live stream from the digital camera. The GUI also enabled the creation of user-defined time-lapse imaging sequences. Each step of a sequence had independent settings for stage position, objective type, and illumination intensity. The chosen sequence would then be repeated at user-defined intervals during the course of an experiment. For example, imaging of the entire growth area was achieved by creating a sequence where the stage position (*x, y, z*) was varied by a constant factor at each step while all other settings were kept constant. The ability to define different settings for each step of a sequence gave the user high flexibility. For instance, the monitoring sequence could include steps with the objective set to a higher magnification for regions of interests in the culture.

Images acquired during time-lapse sequences are either stored for offline analysis or processed online using an embedded MATLAB script node. By using MATLAB for image processing, it gives access to state-of-the-art algorithms, both built in or from the large MATLAB user community. In addition, because the control routine and the image-processing algorithms are separate and compartmentalized, specialists can work on them separately. By embedding MATLAB code directly into LabVIEW, it enables two-way communication between the two and could potentially be used for feedback control of key systems (e.g., fluidics) based on data extracted from imaging. This separation also allows the use of a separate and remote server for image processing so that computationally intensive algorithms do not negatively affect the performance of the LabVIEW control routine.

### Image-Processing Approach

Automated processing of images of cells cultured in the microfabricated bioreactor is made challenging by the nature of the device and the fabrication process but also by biological factors that manifest because of its unique capabilities. The range of cells that are typically observed during a long-term culture is very high and makes it challenging to devise a unique image-processing approach that can accommodate highly varying cell visual features. Moreover, conventional image-processing methods cannot readily accommodate certain experimental scenarios, such as co-culture, which remains the method of choice for the expansion of human embryonic stem cells (hESCs). Fabrication artifacts can also potentially interfere with imaging, for example, scratches on hard polymers (e.g., on the polycarbonate lid of the device). Using conventional image-processing approaches, these artifacts would be detected as cells and would therefore produce inaccurate data.

**Figure 2.**
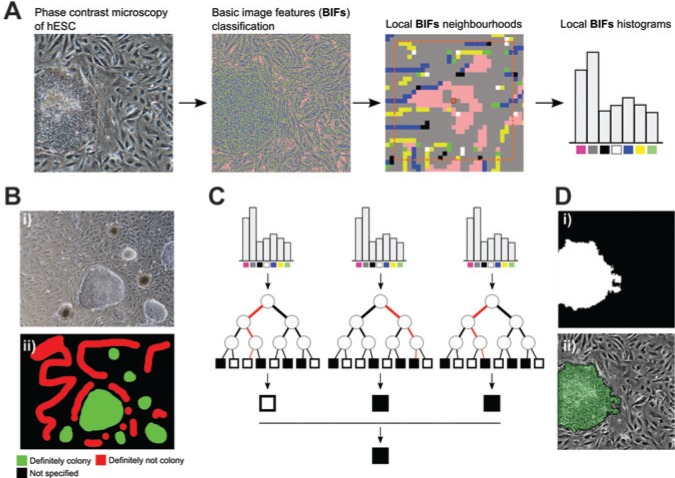
Automated image-processing approach. (**A**) Basic image features (BIFs) of the phase contrast microscopy (PCM) image are first computed. For each pixel, a local histogram of the occurrence of the different BIFs is built. These histograms are the features that are used to classify pixels as background or cells. (**B**) Example of a user-defined training set for the machine learning classifier. Using a conventional image-editing tool, the user indicates portions of an image that are definitely a human embryonic stem cell (hESC) colony and definitely not a colony. It is not necessary to annotate the whole image as regions can be left as not specified. (**C**) Schematic of the random forest classification approach. Local BIF histograms are used as inputs for decision trees. At each node, a binary test based on these features determined whether to traverse to the left or right child node next. A particular tree will classify the histogram as either cell or pixel. The majority vote of multiple trees will decide on the final class assigned to the pixel. (**D**) Example of processing output. (i) Binary mask after processing, showing the stem cell colony in white and the background and fibroblasts in black. (ii) Overlay of the processing results with the original PCM image.

We previously developed an image-processing approach to alleviate these issues to rapidly produce accurate and reliable data, suitable for the monitoring of adherent cell culture in our microfabricated bioreactor.^8^ First, instead of detecting cells based on pixel intensity, we employed BIFs that can be used to classify pixels according to local symmetries.^17^ For example, one of the features was sensitive to dark circular objects on brighter backgrounds and thus often indicated cells’ nuclei. Similarly, the “flat” feature often indicated background regions of the image with a less marked texture. Local histograms of BIFs were constructed for each pixel of the raw PCM image (**Fig. 2A**). A machine learning classifier was then used to classify each pixel of an image based on its associated histogram. To do so, it was first trained by manually annotating regions of the image as either of interest or not (hESC colonies and image background/fibroblast cells, respectively, in **Fig. 2B**). This process is very quick as it is not required to annotate the whole image; instead, ambiguous regions can be left unannotated. This is a key advantage of the method, as image-processing methods often require extensive and tedious parameter tweaking. In this case, the algorithm can be taught how to recognize new cell types in a matter of minutes.

Random forest was chosen as the classifier due to its ability to accommodate very large and noisy data sets such as microscopy images.^9^ In short, it works by building a series of classification trees using a random subset of the training data. At each node of the tree, a random subset of the features available (in our case, bins of the BIF histogram) is used to split the data into one of the classes (in our case, object of interest or rest). Traversing a tree is thus essentially a series of binary tests until the last node (termed *leaf*) is reached and a class is predicted. This is done for a certain number of trees (usually at least 20), and the majority vote is used to decide the final classification (**Fig. 2C**). This process is repeated for each pixel to produce a binary image with the objects of interest in white (value of 1) and the rest (i.e., background and fibroblasts) in black (value of 0) (**Fig. 2D**). This process is quick and robust and, as such, is suitable for monitoring applications.

### Data Derived from Image Processing

**Figure 3.**
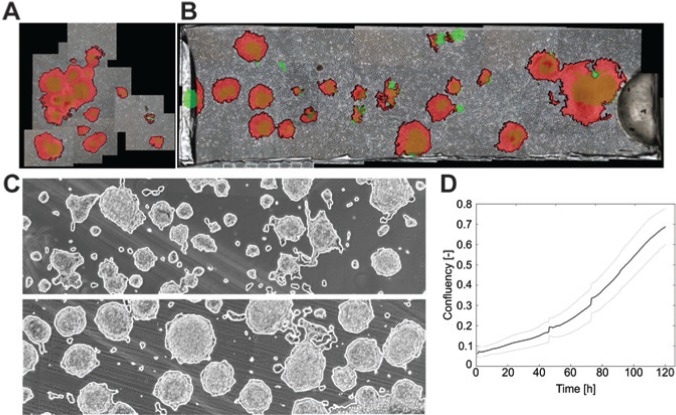
Example of application of image processing to monitor stem cell growth. (**A**) Example of human embryonic stem cells (hESCs) growing in an in vitro fertilization dish. The size of the culture area makes it difficult to quickly image the whole reactor, but instead it is necessary to image only a few fields of view. Green shows colonies on day 1, red on day 3. (**B**) hESCs growing in the microfabricated bioreactor where it is possible to image the whole culture area. Green shows colonies on day 1, red on day 3. (**C**) Application of the same approach to mouse embryonic stem cells cultured in the microfabricated bioreactor. The use of machine learning in combination with basic image features enabled the detection of the cell despite the presence of background artifacts. (**D**) Online monitoring of mESC growth in the whole culture chamber. The bold line is the mean and light gray line the standard deviation across three independent trials.

The ability to detect hESC colonies was first demonstrated using in vitro fertilization (IVF) plates. Due to the relatively large growth area (2.9 cm^2^), only the central area where most of the colonies were seeded could be considered (**Fig. 3A**). By comparing images from day 3 of cultures with those acquired 24 h after seeding, it was possible to assess the growth of the colonies and create striking visual representations of this very dynamic system. The same principle was applied to hESCs growing in the microfabricated bioreactor (**Fig. 3B**). In contrast to the IVF case, the small dimensions of the culture chamber made it possible to monitor growth based on images of the whole culture area. This enabled determining the response of cells to perfusion: colonies were found to migrate, merge, or even wash out on rare occasions. These results were obtained using intermittent imaging. This approach was next applied to fully automated imaging of mESCs cultured in the reactor for long periods (5 days). The results showed that the image-processing method was able to detect mESC colonies accurately despite the prevalence of artifacts (**Fig. 3C**). Based on this detection, the confluency of the culture (i.e., the fraction of the culture area occupied by cells) was determined for the duration of the culture. Interestingly, the mean and standard deviation across three trials were relatively low (26%), demonstrating good reproducibility (**Fig. 3D**).

In conclusion, we have described the integration of a previously described microfabricated cell culture device with an automated image acquisition and processing platform. This was achieved by automating the functions of a microscope and those of a digital camera using custom-developed LabVIEW virtual instruments. A GUI allowed users to easily set up complex time-lapse imaging sequences, for example, to sequentially image the growth area of the microfabricated device. Approaches for offline or online imaging processing using novel machine learning approaches were also presented. The algorithm described enabled the efficient detection of mESCs on unlabeled PCM images. Similarly, the proposed method was able to discriminate between hESC colonies and background fibroblast cells so that the growth of the former could be quantified. Moreover, it was found to be insensitive to common fabrication artifacts and debris. Population growth over the entire culture area was characterized using confluency while tracking of individual cellular objects enabled the detection of growth, death, and migration kinetics.
